# Partitioning of Alkylbenzenes and Aliphatic Alcohols between Hexadecane and Methanol- Water Mixtures

**DOI:** 10.6028/jres.093.012

**Published:** 1988-04-01

**Authors:** Michele (Miller) Schantz, B. N. Barman, Daniel E. Martire

**Affiliations:** National Bureau of Standards Gaithersburg, MD 20899, and Georgetown University, Washington, DC 20057; Georgetown University Washington, DC 20057, and University of Utah Salt Lake City, UT 84112; Georgetown University Washington, DC 20057

**Keywords:** activity coefficients, alcohols, alkylbenzenes, hexadecane, methanol-water mixtures, partition coefficients, reversed phase liquid chromatography

## Abstract

Partition coefficients between *n*-hexadecane and methanol-water mixtures are reported and analyzed for a series of alkylbenzenes and aliphatic alcohols. A custom-designed flask which has a side arm attached near the bottom was used for the measurements. The hexadecane layer was sampled from the top of the flask, and the aqueous layer was sampled through the side arm of the flask. Both phases were analyzed by an appropriate analytical technique, either gas or liquid chromatography, to determine concentrations. A lattice-model theory suggests a correlation between the hexadecane/methanol-water partition coefficients and the solute molar volume for each solute group type and between the hexadecane/methanol-water partition coefficients and the volume fraction of water in the aqueous phase. The mole-fraction-based activity coefficients calculated from the partition coefficients compare favorably to those determined by a headspace gas chromatographic method. Finally, based upon the data contained herein, the retention mechanism in reversed phase liquid chromatography appears to involve the stationary phase as more than just a passive receptor.

## 1. Introduction

Partition coefficients have been measured or estimated for a large number of solutes in an octanol/water system [1]. Recently, the partition coefficients between *n*-hexadecane and water were reported for a series of *n*-alkylbenzenes, *n*-alkanes, *n*-1-alkenes, *n*-1-bromoalkanes, and *n*-1-alcohols [2]. For this paper, the partitioning of *n*-alkylbenzenes and I-alcohols between hexadecane and methanol-water mixtures was studied in an effort to relate this partitioning to retention in reversed phase liquid chromatography (RPLC).

RPLC involves the distribution of a nonpolar or moderately polar solute between a polar mobile phase (the eluent) and a relatively nonpolar stationary phase [3]. The stationary phase is made up of silica gel particles to which *n*-alkyl chains (often, *n*-octadecyl) have been bound. The most common eluents are generally not pure solvents, but rather mixtures of an organic solvent (often, methanol or acetonitrile) and water. When methanol and water are mixed, the solvent properties change [4]. For example, with an increasing percent of methanol in water, the viscosity first increases and then decreases, reaching a maximum at approximately 35% (v/v) methanol in water. The dielectric constant decreases in an almost linear fashion, and the surface tension decreases more rapidly between 0 and 20% (v/v) methanol in water than between 20 and 100% methanol in water.

It has been argued that mobile phase interactions are of principal importance in the control of solute retention, with the nonpolar stationary phase acting mainly as a passive solute acceptor [5]. Many workers in the field (e.g., [6]–[8]) have measured RPLC capacity factors *(k*′) as a function of the volume fraction of organic solvent in water. The capacity factor *(k*′) is given by

$$k^{\prime }=(t_{r}-t_{0})/t_{0},$$ (1)

where *t*_r_, is the retention time of the solute of interest and *t*_0_ is the retention time of an “unretained solute.” The net retention volume, *V*_n_, also has been correlated with the volume fraction of water in the mobile phase [9]. Most of these studies attribute the changes in retention with a change in volume fraction solely to mobile-phase effects.

In the present study, solute partition coefficients have been determined between *n*-hexadecane and methanol-water mixtures for a series of alkylbenzenes and 1-alcohols. In addition, the mutual solubility between hexadecane and methanol has been investigated at 25 °C. Previous partition coefficient measurements done in this laboratory have utilized a generator column attached to an extractor column, a short column packed with C18 material [2]. Since the methanol contained in the aqueous phase would strip the solute from an extractor column, a sit-flask technique similar to that reported by Polak and Lu [10] was used for the present study. The flask had a side arm attached near the bottom which permitted sampling of the aqueous phase without contaminating the syringe with the hexadecane phase. The flask sat undisturbed for 7 days at room temperature, 23 °C. Both phases were analyzed by an appropriate analytical technique, either gas or liquid chromatography. The partition coefficient is the ratio of the concentration in the hexadecane phase to that in the aqueous phase.

As for the hexadecane/water partition coefficient [2], a lattice-model theory suggests a correlation between the hexadecane/methanol-water partition coefficients and the solute molar volume for each solute group type. Using this lattice-model theory, an expression is obtained that relates the hexadecane/methanol-water partition coefficients to the volume fraction of water in the aqueous phase. Finally, the partition coefficients are used in conjunction with chromatographic data to examine the retention mechanism in RPLC.

## 2. Background Thermodynamics

In this treatment, the solute is designated as component a, water as component b, methanol as component b′, and hexadecane as component h. Hence, the partition coefficient of a solute between hexadecane and methanol-water mixtures is denoted by *K*_h/%w_ or *K*_a(h/b′+b)_. In addition, the volume fraction of water in the methanol-water mixtures is denoted by *θ*_b_, and that of methanol by *θ*_b′_. The partition coefficient between hexadecane and methanol-water mixtures is then defined by

$$\ln\;K_{h/\%w}=\ln\;K_{a(h/b^{\prime }+b)}=\ln\;\gamma_{a(b^{\prime }+b)}-\ln\;y_{a(h)},$$ (2)

where *γ*_a_ is the volume-fraction-based infinite-dilution solute activity coefficient, with the convention that *γ*_a_→1 as *θ*_a_→1, where *θ*_a_ is the volume fraction of the solute. Note that eq (2) is analogous to equations for the octanol/water and hexadecane/water partition coefficients [11].

From the Flory-Huggins theory, which is based on a random-mixing (Bragg-Williams) approximation, the solute activity coefficients in water, methanol, and hexadecane are given by [12]

$$\ln\;\gamma_{a(b)}=[1-(r_{a}/r_{b})]+r_{a}X_{\mathrm{ab}}$$ (3)

$$\ln\;\gamma_{a(b^{\prime })}=[1-(r_{a}/r_{b^{\prime }})]+r_{a}X_{\mathrm{ab}\prime }$$ (4)

$$\ln\;\gamma_{a(h)}=[1-(r_{a}/r_{h})]+r_{a}X_{\mathrm{ah}}$$ (5)

In these equations, *r*_i_ is the total number of segments in a molecule of component i which is proportional to $V_{i}*$, the molar volume of component i. Furthermore, *X*_ij_ is the segmental interaction parameter. In the mixed solvent (b+b′), the solute activity coefficient is given by [3]

$$\ln\;\gamma_{a(b+b^{\prime })}=\theta_{b}\ln\;\gamma_{a(b)}+\theta_{b^{\prime }}\ln\;\gamma_{a(b^{\prime })}-r_{a}\theta_{b}\theta_{b^{\prime }}X_{bb^{\prime }}$$ (6)

Substituting eqs (3) and (4) into eq (6) and coupling that result with a substitution of eq (5) into eq (2), one obtains

$$\begin{array}{l} \ln\;K_{a(h/b+b^{\prime })}=r_{a}(1/r_{h}-1/r_{b^{\prime }})\theta_{b^{\prime }}+r_{a}(1/r_{h}-1/r_{b})\theta_{b}\\ \;+r_{a}(X_{ab^{\prime }}-X_{\mathrm{ah}})\theta_{b^{\prime }}+r_{a}(X_{\mathrm{ab}}-X_{\mathrm{ah}})\theta_{b}-r_{a}X_{b^{\prime }b}\theta_{b^{\prime }}\theta_{b}. \end{array}$$ (7)

By definition, the solute, component a, is composed of group type I, the methyl and methylene groups and group type 2, the substituent group. Water, component b, is composed of group type 3, and methanol, component b′, is composed of group type 3′. Hexadecane, component h, is composed of group type 1, the methyl and methylene groups. Expanding eqs (3), (4), and (5) to include the group types and substituting the results into eq (7), the hexadecane/methanol-water partition coefficient is now given by

$$\begin{array}{l} \ln\;K_{a(h/b^{\prime }+b)}=r_{1a}[X_{13^{\prime }}+1/r_{h}-1/r_{b^{\prime }}]\theta_{b^{\prime }}\\ \;+r_{2a}[X_{23^{\prime }}-X_{12}+1/r_{h}-1/r_{b^{\prime }}]\theta_{b^{\prime }}\\ \;+r_{1a}[X_{13}+1/r_{h}-1/r_{b}]\theta_{b}\\ \;+r_{2a}[X_{23}-X_{12}+1/r_{h}-1/r_{b}]\theta_{b}\\ \;-r_{1a}X_{33^{\prime }}\theta_{b^{\prime }}\theta_{b}-r_{2a}X_{33^{\prime }}\theta_{b^{\prime }}\theta_{b} \end{array}$$ (8)

where *r*_ia_ is the number of segments of group type i in the solute molecule (*r*_a_ = *r*_1a_ + *r*_2a_) and where *X*_ij_ is now a group interaction parameter per unit segment.

Knowing the hexadecane/methanol-water partition coefficient at a limited number of volume fractions of water in methanol, *θ*_b_, it is convenient to be able to predict the partition coefficients at other values of *θ*_b_. Neglecting terms involving the product *θ*_b_*θ*_b′_ (*X*_33′_ ≈ 0) and noting that *θ*_b_ + *θ*_b′_ = 1, eq (8) becomes

$$\begin{array}{l} \ln\;K_{a(h/b+b^{\prime })}\approx r_{1a}[X_{13^{\prime }}+1/r_{h}-1/r_{b^{\prime }}]\\ \;+r_{2a}[X_{23^{\prime }}-X_{12}+1/r_{h}-1/r_{b^{\prime }}]\\ \;+r_{1a}[X_{13}-X_{13^{\prime }}+1/r_{b^{\prime }}-1/r_{b}]\theta_{b}\\ \;+r_{2a}[X_{23}-X_{23^{\prime }}+1/r_{b^{\prime }}-1/r_{b}]\theta_{b} \end{array}$$ (9)

Therefore, a plot of ln *K*_a(_*_h/_*_b+b′)_ versus *θ*_b_ would yield a straight line with a slope of *r*_1a_[*X*_13_−*X*_13′_*+*1*/r*_b′_−1*/r*_b_]*+r*_2a_[*X*_23_−*X*_23′_*+*1*/r*_b′_−1*/r*_b_] and an intercept of *r*_1a_[*X*_13′_*+*1/*r*_h_−1/*r*_b′_]+*r*_2a_[*X*_23′_−*X*_12_+1/*r*_h_−1*/r*_b'_].

It is also convenient to correlate the *K*_a(h/b+b′)_ at a particular *θ*_b_ with the molar volume of the solute $V_{a}*$. In other words, if one knows the *K*_a(h/b+b′)_ for several members of a solute series at a particular *θ*_b_, one may wish to predict that partition coefficient for another member of that series, knowing its molar volume. Using eq (9) with *r*_1a_.*= r*_a_−*r*_2a_ one finds that

$$\begin{array}{l} \ln\;K_{a(h/b+b^{\prime })}\approx r_{a}[X_{13^{\prime }}+1/r_{h}-1/r_{b^{\prime }}]\\ \;+r_{2a}[X_{23^{\prime }}-X_{12}-X_{13^{\prime }}]\\ \;+r_{a}[X_{13}-X_{13^{\prime }}+1/r_{b^{\prime }}-1/r_{b}]\theta_{b}\\ \;+r_{2a}[X_{23}-X_{23^{\prime }}-X_{13}+X_{13^{\prime }}]\theta_{b}. \end{array}$$ (10)

However, *r*_a_ is proportional to $V_{a}*$, the molar volume of the solute and *r*_2a_ is proportional to $V_{2a}*$, the molar volume of the solute functional group. The partition coefficient can now be rewritten as

$$\begin{array}{l} \ln\;K_{a(h/b+b^{\prime })}=CV_{a}*\;[X_{13^{\prime }}+1/r_{h}-1/r_{b^{\prime }}]\\ \;+CV_{a}*\;[X_{13}-X_{13^{\prime }}+1/r_{b^{\prime }}-1/r_{b}]\theta_{b}\\ \;+CV_{2a}*\;[X_{23^{\prime }}-X_{12}-X_{13^{\prime }}]\\ \;+CV_{2a}*[X_{23}-X_{23^{\prime }}-X_{13}+X_{13^{\prime }}]\theta_{b}, \end{array}$$ (11)

where *C* is a constant. This equation predicts a linear relationship between In *K*_a(h/b+b′)_ and $V_{a}*$, where the slope is *C*[*X*_13′_*+*1*/r*_h_−1*/r*_b_]*+C*[*X*_13_−*X*_13′_*+*1*/r*_b′_−1/r_b_]*θ*_b_ and the intercept is $CV_{2a}*[X_{23^{\prime }}-X_{12}-X_{13^{\prime }}]+CV_{2a}*\;[X_{23}-X_{23^{\prime }}-X_{13}+X_{13^{\prime }}]\theta_{b}$. Therefore, according to eq (11), at a given *θ*_b_ the slope should be independent of the solute functional group, whereas, the intercept should reflect the size and interactions of the functional group. Similar linear relationships between the octanol/water partition coefficient, *K*_o/w_, and $V_{a}*$, as well as the hexadecane/water partition coefficient, *K*_h/w_, and $V_{a}*$, were discussed in a previous paper [2].

## 3. Experimental

A sit flask was used for the partition coefficient measurements. The solute was quantitatively added to the hexadecane phase. The aqueous layer was placed at the bottom of a flask containing a side arm. The hexadecane-solute mixture was also added to the flask. This flask was then allowed to sit in a room kept at 23 °C without stirring for at least 7 days [10]. The propylbenzene in hexadecane*/*100% methanol partition coefficient (*K*_h/0%w_) was used as the test system. Samples of both layers were removed and analyzed after 3, 5, 7, 9, and 11 days of sitting. The *K*_h/0%w_ value was constant within experimental error from day 7 to day 11.

Both phases were analyzed by an appropriate analytical method, either gas or liquid chromatography. The hexadecane layer was sampled through the top of the sit flask while the aqueous layer was sampled through the side arm.

## 4. Results and Discussion

The mole fraction of methanol in *n*-hexadecane at 25 °C is 3 × 10^−3^ mole hexadecane per total number of moles in the methanol phase, and the mole fraction of *n*-hexadecane in methanol at 25 °C is 4.25 × 10^−2^ mole methanol per total number of moles in the hexadecane phase. The results for the partition coefficients between hexadecane and methanol-water mixtures are given in tables 1, 2, and 3 for the alcohols, benzene, and alkylbenzenes, respectively. The data revealed some dependence of the partition coefficient between hexadecane and methanol-water mixtures on concentration of the alcohol in the hexadecane phase for the alcohols studied. Riebesehl and Tomlinson [13] noted that, although alcohols tend to self-associate in nonaqueous solution, association below 10^−2^ mol/L is extremely small. Backlund et al. [14] measured the partition coefficients of alcohols between octane and alcohol-water mixtures. They noted a concentration dependence of the partition coefficient indicating self-association of the alcohols in the octane phase. In the case of the alkylbenzenes, a clear dependence on concentration was found for the partition coefficients, especially between hexadecane and 100% methanol (*K*_h/0%w_), suggesting aggregation effects. As Karickhoff and Brown [15] suggested, the monomer partition coefficient is determined by reducing the solute concentration below the aggregation threshold concentration, experimentally determined by consecutive dilutions. Association generally tends to raise the partition coefficient, and dissociation tends to lower the partition coefficient [16]. In the case of benzene and the alkylbenzenes, with increasing aromatic concentration in the hexadecane phase, the hexadecane/methanol partition coefficient first increases. For the alkylbenzenes, the *K*_h/0%w_ levels off at this point. For benzene, however, *K*_h/0%w_ next reaches a minimum and then increases again. In all cases (alcohols, benzene, and alkylbenzenes), the lowest value of *K*_h/0%w_ determined was taken as the “true” value.

There are only three sets of literature values of any similarity to those reported here, two of which are those of Krustulovic et al. [17] and Lochmuller and Wilder [18] who determined the liquid-liquid extraction between *n*-hexadecane and a 90% acetonitrile in water mixture for hexane and octane or a 50% methanol in water mixture for benzene and toluene, respectively. They determined the relative partition coefficients in the hexadecane layer, log a, however. The other set are preliminary data of Hussam and Carr [19] who determined the infinite dilution activity coefficients of benzene, toluene, ethylbenzene, *n*-propylbenzene, and *n*-butylbenzene in methanol-water mixtures at 30 °C by a headspace gas chromatographic method. They studied solutes at concentrations as low as 10^−6^ mole fraction up to 10^−3^ mole fraction to insure that solute self-association was negligible. The polynomial equations obtained from their data relating the mole-fraction-based activity coefficients to the mole fraction of water in methanol are given in table 4.

In a previous publication [2], the volume-fraction-based activity coefficients in hexadecane as determined from a gas-liquid chromatographic experiment are reported. Using the relationships for activity (*a*) of a solute, one can relate the activity coefficient on a volume-fraction basis (γ_a,_*_θ_*) to that on a mole-fraction basis (γ_a,_*_x_*), i.e.

$$a=\gamma_{a,\theta }\theta_{a}=\gamma_{a,x}x_{a},$$ (12)

where *θ_a_* and *x_a_* are, respectively, the volume fraction and mole fraction of the solute in solution. From eq (12), it follows that

$$\gamma_{a(h),x}\approx \gamma_{a(h),\theta }(V_{a}*/V_{h}*)$$ (13)

where $V_{a}*$ and $V_{h}*$ are the molar volumes of the solute and hexadecane, respectively. Using the γ_a(h),_*_θ_* reported previously [2] and eq (13), the γ_a(h),_*_x_* are calculated for the alkylbenzene series, benzene through butylbenzene as reported in table 5. From ln *K*_h/0%w_ and γ_a(h),_*_x_* the mole-fraction-based activity coefficients at the corresponding mole fraction of water in methanol are calculated using eq (2) (table 5). Using the equations obtained by fitting Hussam and Carr’s data (table 4), the infinite dilution mole-fraction-based activity coefficients were determined at the same mole fractions of water in methanol as in the partitioning experiments. The data are presented in table 5. Note that the agreement between the activity coefficients determined at infinite dilution and from partition coefficients is fairly good. At 100% water, the activity coefficients determined from aqueous solubilities are also given. There is a relatively good agreement among all three activity coefficients for benzene. For *n*-butylbenzene, an extrapolation of Hussam and Carr's data [19] from *x*_b_=0.53 to *x*_b_ ≈1.00, where *x*_b_ denotes the mole fraction of water in the water-methanol mixture, is required to obtain a value at 100% water.

**Figure 1:**
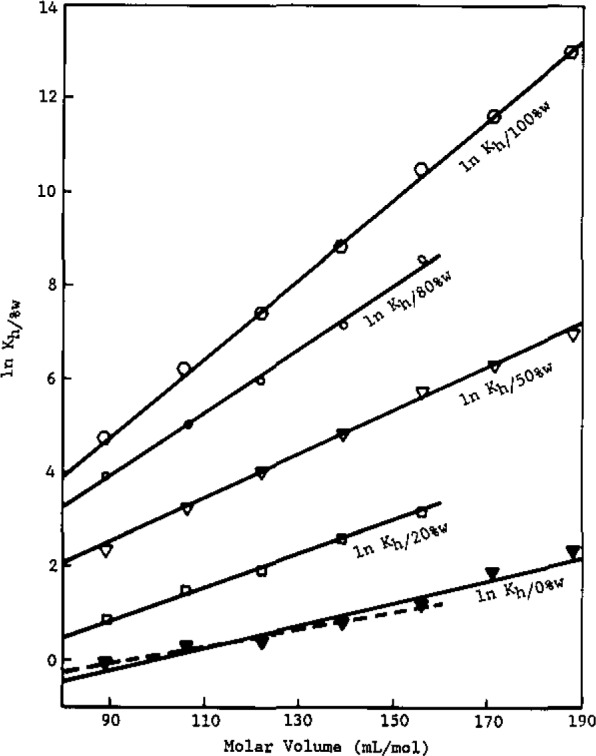
ln *K*_h/%w_ versus molar volume for the alkylbenzenes.

**Figure 2:**
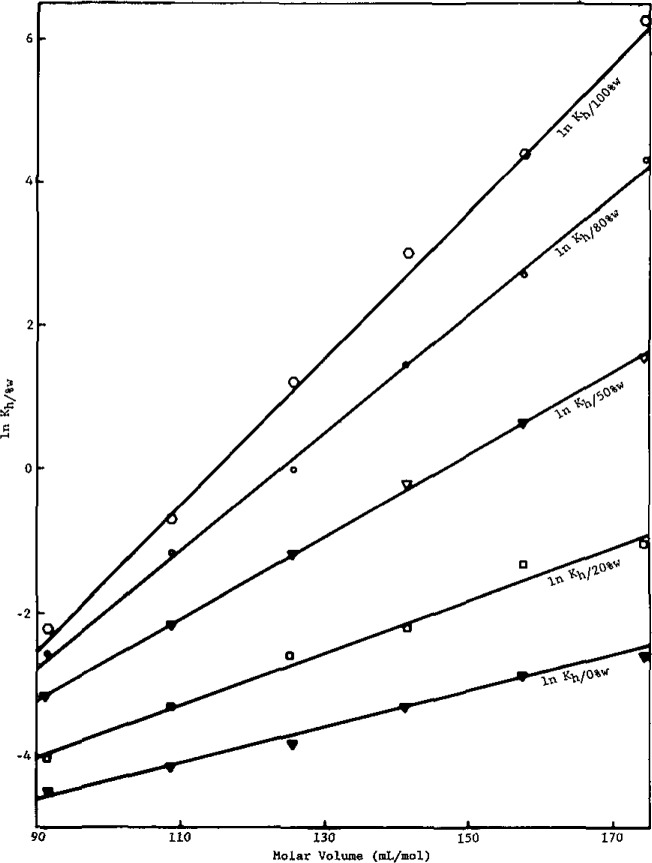
ln *K*_h/%w_ versus molar volume for the alcohols.

The hexadecane/methanol-water partition coefficient, similar to the hexadecane/water and octanol/water partition coefficients [2], is linearly related to the solute molar volnme by eq (11). As can be seen in figures 1 and 2 for the alkylbenzenes and alcohols, respectively, there is a linear relationship between ln *K*_h/0%w_ and solute molar volume at a particular volume percent for the solutes studied. The volume percents of water in methanol used are 0%, 20%, 50%, 80%, and 100% water in methanol.

In figure 1, equations for each percent of water in methanol are:

$$\begin{array}{l} \ln\;K_{h/100\%w}=0.0831(\pm 0.0018)V_{a}*-2.67(\pm 0.05),\\ \;r^{2}=0.999(\pm 0.019)\\ \ln\;K_{h/80\%w}=0.0676(\pm 0.0009)V_{a}*-2.17(\pm 0.03),\\ \;r^{2}=0.995(\pm 0.015)\\ \ln\;K_{h/50\%w}=0.0467(\pm 0.0011)V_{a}*-1.71(\pm 0.05),\\ \;r^{2}=0.996(\pm 0.021)\\ \ln\;K_{h/20\%w}=0.0353(\pm 0.0009)V_{a}*-2.33(\pm 0.06),\\ \;r^{2}=0.997(\pm 0.022)\\ \ln\;K_{h/0\%w}=0.0241(\pm 0.0008)V_{a}*-2.39(\pm 0.08),\\ \;r^{2}=0.956(\pm 0.019) \end{array}$$

Deleting *n*-pentylbenzene and *n*-hexylbenzene,

$$\begin{array}{l} \ln\;K_{h/0\%w}=0.0716(\pm 0.0010)V_{a}*-1.66(\pm 0.05),\\ \;r^{2}=0.978(\pm 0.021). \end{array}$$

The values in the parentheses indicate the standard deviation of the slope, intercept, and overall fit, respectively, for each equation.

In figure 2, equations for each percent of water in methanol are:

$$\begin{array}{l} \ln\;K_{h/100\%w}=0.103(\pm 0.004)V_{a}*-11.74(\pm 0.09),\\ \;r^{2}=0.998(\pm 0.015)\\ \ln\;K_{h/80\%w}=0.082(\pm 0.003)V_{a}*-10.17(\pm 0.10),\\ \;r^{2}=0.997(\pm 0.018)\\ \ln\;K_{h/50\%w}=0.057(\pm 0.006)V_{a}*-8.40(\pm 0.10),\\ \;r^{2}=0.999(\pm 0.017)\\ \ln\;K_{h/20\%w}=0.037(\pm 0.004)V_{a}*-7.35(\pm 0.09),\\ \;r^{2}=0.991(\pm 0.020)\\ \ln\;K_{h/0\%w}=0.024(\pm 0.003)V_{a}*-6.79(\pm 0.08),\\ \;r^{2}=0.993(\pm 0.019). \end{array}$$

The worst correlation coefficient is for the alkylbenzene partition coefficients in 100% methanol, where as seen in tables 2 and 3, there is considerable dependence of these values on the concentration of alkylbenzenes in the hexadecane phase, making it difficult to determine the “true” infinite-dilution partition coefficient.

One notes in eq (11) that both the slope and intercept of plots of ln *K*_h/%w_ versus $V_{a}*$ depend on the volume fraction of water in methanol, *θ*_b_. As can be seen in figures 1 and 2, as *θ*_b_ increases the slopes increases for a given homologous series. Also, the slopes are approximately the same at a given *θ*_b_ for the alkylbenzene and alcohol series. However, the intercepts are highly negative for the alcohols, becoming more so with increasing *θ*_b_, while they are much less negative and essentially independent of *θ*_b_ for the alkylbenzenes.

**Figure 3:**
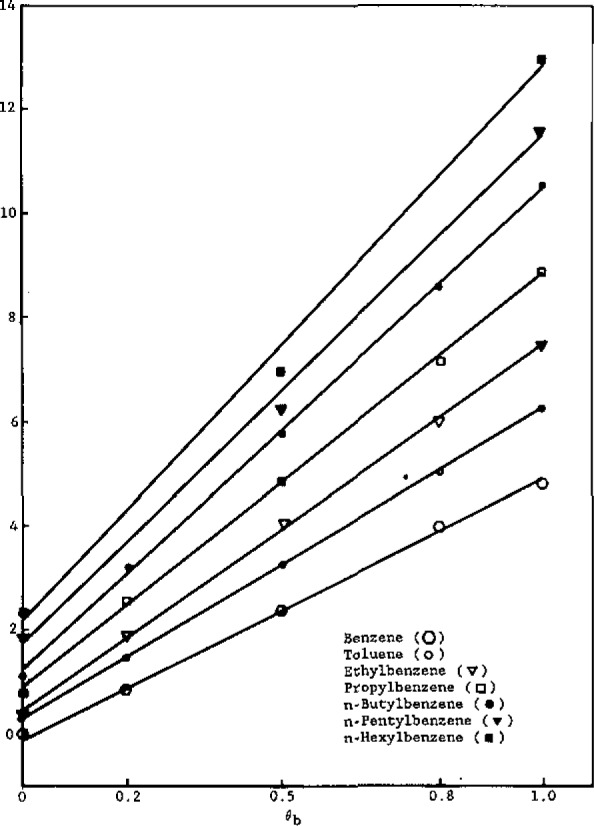
ln *K*_h/0%w_ versus volume fraction of water in methanol for the alkylbenzenes.

**Figure 4:**
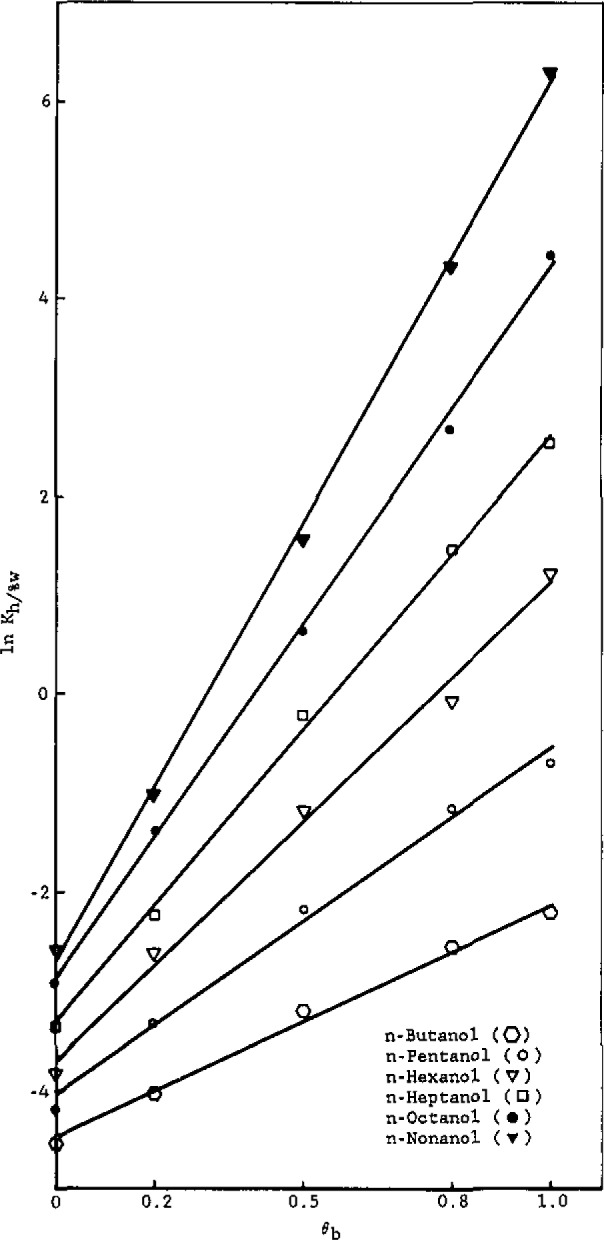
ln *K*_h/0%w_ versus volume fraction of water in methanol for the alcohols.

To interpret these trends, let us first examine the dependence of the partition coefficients on *θ*_b_, shown in figures 3 and 4 for the alkylbenzenes and alcohols, respectively.

In figure 3, equations of the lines are:

$$\begin{array}{l} \text{Benzene}\;\ln\;K_{h/\%w}=4.94(\pm 0.06)\theta_{b}-0.09(\pm 0.01),\\ \;r^{2}=0.998(\pm 0.021)\\ \text{Toluene}\;\ln\;K_{h/\%w}=5.93(\pm 0.08)\theta_{b}+0.30(\pm 0.01),\\ \;r^{2}=0.999(\pm 0.016)\\ \text{Ethylbenzene}\;\ln\;K_{h/\%w}=6.95(\pm 0.09)\theta_{b}\\ \;+0.47(\pm 0.01),\;r^{2}=0.999(\pm 0.019)\\ \text{Propylbenzene}\;\ln\;K_{h/\%w}=7.91(\pm 0.06)\theta_{b}\\ \;+0.89(\pm 0.01),\;r^{2}=0.999(\pm 0.019)\\ n-\text{Butylbenzene}\;\ln\;K_{h/\%w}=9.23(\pm 0.08)\theta_{b}\\ \;+1.22(\pm 0.02),\;r^{2}=0.999(\pm 0.021)\\ n-\text{Pentylbenzene}\;\ln\;K_{h/\%w}=9.75(\pm 0.09)\theta_{b}\\ \;+1.70(\pm 0.02),\;r^{2}=0.997(\pm 0.024)\\ n-\text{Hexylbenzene}\;\ln\;K_{h/\%w}=10.59(\pm 0.11)\theta_{b}\\ \;+2.13(\pm 0.03),\;r^{2}=0.995(\pm 0.022). \end{array}$$

In figure 4, equations of the lines are:

$$\begin{array}{l} n-\text{Butanol}\;\ln\;K_{h/\%w}=2.36(\pm 0.04)\theta_{b}\\ \;-4.48(\pm 0.06),\;r^{2}=0.993(\pm 0.021)\\ n-\text{Pentanol}\;\ln\;K_{h/\%w}=3.50(\pm 0.05)\theta_{b}\\ \;-4.06(\pm 0.06),\;r^{2}=0.993(\pm 0.019)\\ n-\text{Hexanol}\;\ln\;K_{h/\%w}=4.87(\pm 0.07)\theta_{b}\\ \;-3.74(\pm 0.08),\;r^{2}=0.994(\pm 0.018)\\ n-\text{Heptanol}\;\ln\;K_{h/\%w}=5.93(\pm 0.06)\theta_{b}\\ \;-3.33(\pm 0.07),\;r^{2}=0.998(\pm 0.016)\\ n-\text{Octanol}\;\ln\;K_{h/\%w}=7.18(\pm 0.09)\theta_{b}9\\ \;-2.90(\pm 0.08),\;r^{2}=0.999(\pm 0.019)\\ n-\text{Nonanol}\;\ln\;K_{h/\%w}=8.88(\pm 0.08)\theta_{b}\\ \;-2.73(\pm 0.06),\;r^{2}=0.999(\pm 0.017). \end{array}$$

Workers [6,8] have studied the dependence of the RPLC capacity factor (*k′*) on the volume fraction of water in methanol. Hennion et al. [6] found a linear relation between ln *k′* and *θ*_b_, while other workers [8] contend that these plots are linear for only part of the aqueous volume fraction range. Theoretical justification for a linear relationship between ln *K*_h/%w_ and *θ*_b_ is given by eq (9), which reveals that both the intercept and slope depend on *r*_1a_, the number of alkyl segments in the solute molecule.

To analyze figures 3 and 4 in terms of eq (9), let us first assign one segment to each methylene group. Accordingly, from relative van der Waals volumes [20], a methyl group would correspond to 1.33 segments, a bound hydrogen atom to approximately 0.33 segments, and a phenyl group to 4.48 segments. Thus, *r*_b_= 1.00 (water), r_b′_ =2.00 (methanol), *r*_h_= 16.67 (hexadecane), and, for the solutes,

$$\text{alkylbenzenes}:\;r_{a}=4.48+(n_{a}+0.33)$$ (14)

$$\text{alcohols}:\;r_{a}=0.67+(n_{a}+0.33),$$ (15)

where *n*_a_ is the number of carbon atoms in the solute *n*-alkyl chain, *r*_1a_ *= n*_a_*+*0.33, and *r*_2a_ is 4.48 and 0.67 for the phenyl and hydroxyl groups, respectively.

Using these assigned volumes and eq (9) a linear least-squares analysis of the coefficients of the *θ*_b_ term for alkylbenzenes (fig. 3) as a function of *r*_1a_ gives *X*_23_−*X*_23′_.= 1.56 and *X*_13_−*X*_13′_.= 1.45, with a correlation coefficient of 0.997. These results indicate that, in terms of interaction energetics, both the phenyl group and alkyl groups prefer to dissolve in methanol rather than water. Similarly, analysis of the intercepts of the plots (fig. 3) as a linear function of *r*_1a_ yields *X*_23′_−*X*_12_=0.38 and *X*_13′_=0.80 (whence, *X*_13_=2.25), with a correlation coefficient of 0.994. The former result indicates that, energetically, phenyl groups prefer alkyl groups over water, while the latter result indicates that methanol (and especially water) are not hospitable solvents for alkyl groups. A linear least-squares analysis of the coefficients of the *θ*_b_ term for the alcohols (fig. 4) as a function of *r*_1a_ gives *X*_23_−*X*_23′_ = −4.41 and *X*_13_−*X*_13′_ = 1.78, with a correlation coefficient of 0.998. The former result clearly indicates that, as expected, hydroxyl groups energetically prefer solution in water over solution in methanol. The latter result (already discussed) is somewhat higher than that obtained from analysis of the alkylbenzene plots (1.45); however, in view of the approximate nature of the model and the uncertainty in the experimental data, the agreement is reasonable. Similarly, analysis of the intercepts of the plots (fig. 4) as a linear function of *r*_1a_ yields *X*_23′_−*X*_12_ = −8.57 and *X*_13′_ =0.80 (in full agreement with the alkylbenzene result), with a correlation coefficient of 0.995. The former value reflects the very strong energetic preference of hydroxyl groups for dissolving in methanol, relative to solution in an alkyl-group environment.

Returning to the interpretation of figures 1 and 2, note that all of the molecular parameters in eq (11) have now been assigned (*r* values) or determined *(X* values). Inserting these values into eq (11), one obtains for the alkylbenzenes and alcohols, respectively:

$$\begin{array}{l} \ln\;K_{a(h/b+b^{\prime })}=V_{a}*C[0.36+0.95\theta_{b}]\\ \;+V_{2a}*C[-0.42+0.11\theta_{b}] \end{array}$$ (16)

$$\begin{array}{l} \ln\;K_{a(h/b+b^{\prime })}=V_{a}*C[0.36+1.28\theta_{b}]\\ \;+V_{2a}*C[-9.37-6.19\theta_{b}]. \end{array}$$ (17)

As discussed earlier and as can be seen by comparing eqs (16) and (17), the coefficients of the $V_{a}*$ term are approximately the same for the alkylbenzene and alcohol series, and they increase with increasing *θ*_b_, primarily reflecting the unfavorable mixing of alkyl groups with water, relative to their mixing with methanol (*X*_13_−*X*_13′_).

For the alkylbenzenes, the intercepts in figure 1 are negative and are virtually independent of *θ*_b_. This can be understood by examining the $V_{2a}*$ term in eq (16) in the light of eq (11). The lack of a discernible dependence of these intercepts on *θ*_b_ reflects the near cancellation of four *X* terms in eq (11), i.e., *X*_23_−*X*_23′_−*X*_13_+*X*_13′_≈0. The negative intercepts stem primarily from the relative preference of methanol for phenyl groups over alkyl groups, i.e., *X*_23′_−*X*_13_<0. For the alcohols [eq (17)], the intercepts in figure 2 are highly negative and become even more negative with increasing *θ*_b_. When *θ*_b_ =0 (pure methanol), the intercept is proportional to *X*_23′_−*X*_12_−*X*_13′_ (= −9.37). Clearly, the very negative *X*_23′_−*X*_12_, reflecting the solution preference of hydroxyl groups for methanol rather than alkyl groups, governs here. With increasing *θ*_b_ both the preference of hydroxyl groups for water over methanol (*X*_23_−*X*_23′_=−4.41) and of alkyl groups for methanol over water (*X*_13′_−*X*_13_= −1.78) lead to more negative intercepts. Note that even though $V_{2a}*$ for the hydroxyl group is smaller than that of the phenyl group by a factor of about 6.7 (from the ratio of *r*_2a_ values), the coefficient of the $V_{2a}*$ term in eq (17) is more negative by a factor of 22 (*θ*_b_=0) to 50 (*θ*_b_ = 1) than the corresponding coefficient in eq (16).

Finally, we address the important question as to whether the entire dependence of solute retention on mobile-phase composition can be attributed solely to the mobile phase or whether the stationary-phase contribution varies with mobile-phase composition also. Assuming that in RPLC systems the solute partitions between two “bulk” phases, the net retention volume is given by

$$V_{n}=K_{a(s/b+b^{\prime })}C_{s},$$ (18)

where *K*_a(s/b+b′)_ is the partition coefficient of the solute between the mobile phase and stationary phase and *C*_s_ is the capacity of the stationary phase, given here by *V*_s_, the volume. The partition coefficient is the ratio of the volume-fraction-based activity coefficients such that

$$K_{a(s/b+b^{\prime })}=\gamma_{a(b+b^{\prime })}/\gamma_{a(s)},$$ (19)

where γ_a(s)_ is the apparent activity coefficient of the solute in the stationary phase. Therefore, substituting eq (19) into eq (18) and using eq (2), the hexadecane/methanol-water partition coefficient (*K*_a(h/b+b′)_*=K*_h/%w_) divided by *V*_n_ is given by

$$\ln[K_{a(h/b+b^{\prime })}/V_{n}]=\ln[\gamma_{a(s)}/\gamma_{a(h)}]-\ln\;C_{s}.$$ (20)

**Figure 5:**
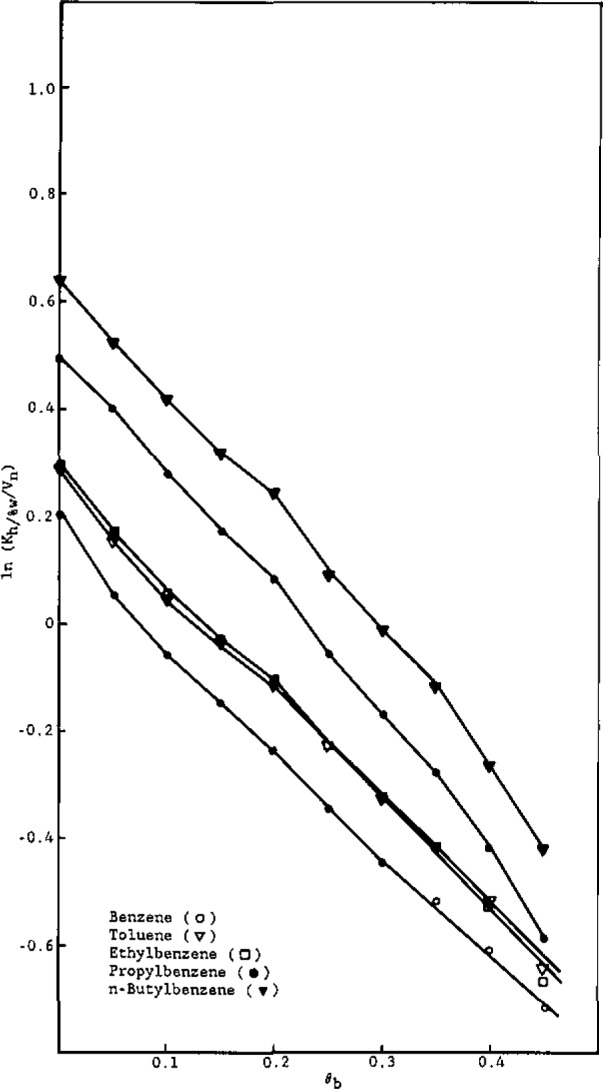
Relationship between ln (*K*_h/%w_/*V*_n_) and *θ*_b_ for the alkylbenzenes.

**Figure 6:**
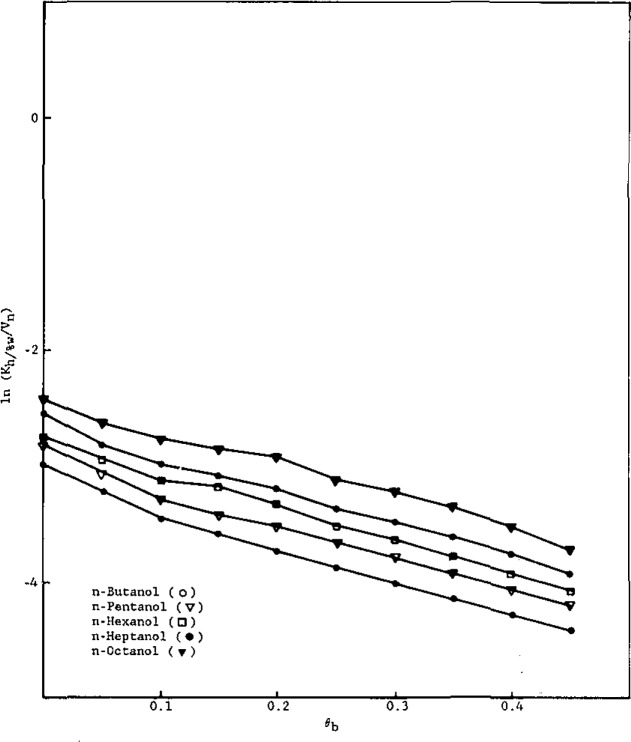
Relationship between ln (*K*_h/%w_/*V*_n_) and *θ*_b_ for the alcohols.

Using *V_n_* determined on a Zorbax ODS column at 25 °C [9], the plots of ln [*K*_h/%w_/*V*_n_] versus *θ*_b_ the volume fraction of water in methanol, are shown in figures 5 and 6 for the alkylbenzenes and alcohols, respectively. It is observed that ln [*K*_h/%w_/*V*_n_] decreases with increasing *θ*_b_, for the solutes studied.

Again we are assuming that the RPLC retention is governed by a single mechanism involving solute partitioning between the mobile phase and the stationary phase consisting of the bonded C18 chains and any solvent (methanol and/or water) which is absorbed by the chains. Examining eq (20), one expects that *C*_s_ should decrease with increasing *θ*_b_, since any solvent uptake decreases with increasing *θ*_b_. This, however, leads to an increase in ln [*K*_h/%w_/*V*_n_]. Therefore, γ_a(s)_ must decrease dramatically with increasing *θ*_b_. The solute may be experiencing a mixed C18+methanol environment with pure methanol (*θ*_b_ =0). Presumably, the methanol is gradually expelled from the stationary phase with increasing *θ*_b_ leading to a more favorable environment thus the decrease in γ_a(s)._ In any event, the stationary phase contribution to *V*_n_ is not independent of *θ*_b_.

Another possible interpretation is that RPLC retention is governed by some multiple mechanism where the relative contributions vary with *θ*_b_. Considering a dual mechanism (s and t),

$$V_{n}=K_{a(s/b+b^{\prime })}C_{s}+K_{a(t/b+b^{\prime })}C_{t}$$ (21)

Here, s might refer to contributions from solute absorption (solvation) by the C18 chains plus a limited amount of incorporated methanol, and t might refer to the contribution from solute adsorption/displacement at the mobile phase-bonded phase interface. At the interface, the composition of the relevant interfacial layer would vary with *θ*_b_ and is richer in methanol than the mobile phase. *C*, is then the volume of the bonded phase with any solvent uptake included, and *C*, is the volume of the inter-facial layer which may vary with varying *θ*_b_. Therefore, the partition coefficients are given by

$$K_{a(s/b+b^{\prime })}=\gamma_{a(b+b^{\prime })}/\gamma_{s}$$ (22)

$$K_{a(t/b+b^{\prime })}=\gamma_{a(b+b^{\prime })}/\gamma_{t},$$ (23)

where γ_a(s)_ and γ_a(t)_ are the apparent solute activity coefficients in the stationary phase for retention modes s and t, respectively. Now the hexadecane/methanol-water partition coefficient divided by the net retention volume is given by

$$\begin{array}{l} \ln[K_{a(h/b+b^{\prime })}/V_{n}]=-\ln[C_{s}\gamma_{a(h)}/\gamma_{a(s)}]\\ \;-\ln[1+(\gamma_{a(s)}C_{t}/\gamma_{a(t)}C_{s})]. \end{array}$$ (24)

Looking at eq (24), one could argue that *C*_s_/γ_a(s)_ should remain fairly constant as *θ*_b_ increases since there should be little uptake of solvent within the CI8 chains. Therefore, the observed decrease in ln [*K*_h/%w_/*V*_n_] with increasing *θ*_b_ would then be due to an increase in (γ_a(s)_*C*_t_)/(γ_a(t)_*C*_s_) suggesting that γ_a(t)_/*C*_t_, primarily γ_a(t)_ must decrease with increasing γ_b_' Possibly, as lib increases, the interface becomes less rich in methanol so the solute can more readily displace the adsorbed solvent, thus lowering γ_a(t)_.

From these observations, it is clear that regardless of the retention mechanism, single or multiple, the stationary phase *is* not a “passive receptor.” It appears to become progressively more hospitable to the solute with increasing *θ*_b_.

## Figures and Tables

**Table 1:** Dependence of the bexadecane/metbanol-water partition coefficient on concentration in bexadecane and percent methanol in the aqueous phase for the alcohols

Compound	100% H_2_O		80% H_2_O		50% H_2_O		20% H_2_O		0% H_2_O	
	[in *C*_16_](M)	ln *K*_h/100%w_^a^	[in *C*_16_](M)	ln *K*_h/80%w_^a^	[in *C*_16_](M)	ln *K*_h/50%w_^a^	[in *C*_16_](M)	ln *K*_h/20%w_^a^	[in *C*_16_](M)	ln *K*_h/0%w_^a^
1-Butanol	1.04 × 10^−1^	−2.22±0.09	1.209×10^−1^	−2.56±0.01	0.69×10^−1^	−2.95±0.06	0.69×10^−1^	−4.05±0.10	1.19×10^−1^	−4.51±0.01
	0.16×10^−1^	−2.21±0.06	0.16×10^−1^	−2.55±0.02	0.16×10^−1^	−3.18±0.08	0.16×10^−1^	−4.04±0.05	0.16×10^−1^	−4.52±0.04
1-Pentanol	1.20×10^−1^	−0.07±0.01	1.13×10^−1^	−1.16±0.02	0.59 × 10^−1^	−2.17±0.02	0.59×10^−1^	−3.32±0.06	0.59×10^−1^	−4.19±0.02
	0.12×10^−1^	−0.71±0.03	0.12×10^−1^	^−^1.16±0.03	0.12×10^−1^	−2.19±0.05	0.12×10^−1^	−3.32±0.04	0.12×10^−1^	−4.18±0.06
1-Hexanol	0.67×10^−1^	1.23±0.01	1.11×10^−1^	−0.77±0.01	0.87×10^−1^	−1.20±0.04	0.87×10^−1^	−2.23±0.07	0.87×10^−1^	−3.38±0.07
	0.40 ×10^−1^	1.23±0.03	0.40×10^−1^	−0.08±0.01	0.40×10^−1^	−1.21±0.03	0.40×10^−1^	−2.62±0.05	0.40×10^−1^	−3.88±0.02
			0.15×10^−1^	−0.07±0.01			0.15×10^−1^	−2.64±0.03	0.15 ×10^−1^	−3.85±0.05
1-Heptanol	0.46×10^−1^	2.53±0.08	1.27±10^−1^	1.97±0.09	1.17×10^−1^	−0.75±0.01	1.17×10^−1^	−2.62±0.08	1.17±10^−1^	−3.34±0.01
	0.16×10^−1^	2.51±0.03	0.16×10^−1^	1.46±0.02	0.16 ×10^−1^	−0.24±0.01	0.16×10^−1^	−2.23±0.04	0.16×10^−1^	−3.34±0.05
			0.06×10^−1^	1.45±0.05	0.06×10^−1^	−0.21±0.02	0.06×10^−1^	−2.24±0.02		
1-Octanol	0.90×10^−1^	4.42±0.01	1.03×10^−1^	2.17±0.02	0.91 ×10^−1^	1.02±0.02	0.91×10^−1^	−2.07±0.01	1.03×10^−1^	−2.58±0.05
	0.16×10^−1^	4.40±0.04	0.16×10^−1^	2.67±0.03	0.16×10^−1^	0.62±0.01	0.16×10^−1^	−1.40±0.02	0.16×10^−1^	−2.95±0.04
			0.05×10^−1^	2.69±0.03	0.05×10^−1^	0.63±0.02	0.05×10^−1^	−1.39±0.04	0.05×10^−1^	−2.91±0.05
1-Nonanol	0.57×10^−1^	6.29±0.01	0.96×10^−1^	3.59±0.09	1.22 ×10^−1^	1.98±0.02	1.22×10^−1^	−1.46±0.03	0.96×10^−1^	−2.58±0.09
	0.09 ×10^−1^	6.28±0.02	0.09×10^−1^	4.29±0.05	0.09×10^−1^	1.57±0.01	0.09×10^−1^	−1.04±0.02	0.09×10^−1^	−2.59±0.05
			0.06×10^−1^	4.31±0.04	0.06×10^−1^	1.56±0.02	0.06×10^−1^	−1.04±0.03		

a The uncertainty is the standard deviation of the mean for three replicate measurements.

**Table 2:** Dependence of the hexadecane/methanol-water partition coefficient (*K*_h/%w_) on concentration in hexadecane and percent methanol in the aqueous phase for benzene

% H_2_O	[Benzene in *C*_16_](M)	ln *K*_h/%w_
100	9.90×10^−2^	4.77±0.01
80	9.29×10^−2^	4.21±0.01
	0.02×10^−2^	3.99±0.05
50	9.29×10^−2^	2.72±0.01
	1.35×10^−2^	3.10±0.04
	1.14×10^−2^	2.65±0.03
	0.99×10^−2^	2.34±0.02
	0.02×10^−2^	2.37±0.05
40	0.99×10^−2^	1.84±0.02
30	0.99×10^−2^	1.46±0.02
20	9.29×10^−2^	1.10±0.02
	0.99×10^−2^	0.99±0.02
	0.02×10^−2^	0.82±0.01
10	0.99×10^−2^	0.41±0.01
0	9.29×10^−2^	−0.41±0.01
	6.88×10^−2^	−0.78±0.01
	2.36×10^−2^	−1.05±0.02
	0.92×10^−2^	−0.42±0.01
	0.64×10^−2^	−0.27±0.02
	0.24×10^−2^	−0.08±0.01
	0.23×10^−2^	−0.09±0.01
	0.21×10^−2^	−0.10±0.02
	0.13×10^−2^	−0.11±0.01
	0.11×10^−2^	−0.07±0.01
	0.10×10^−2^	−0.07±0.01
	0.03×10^−2^	−0.13±0.01
	0.01×10^−2^	−0.16±0.01

a The uncertainty is the standard deviation of the mean for three replicate measurements.

**Table 3:** Dependence of the hexadecane/methanol-water partition coefficients on concentration in hexadecane and percent methanol in the aqueous phase for biphenyl and the alkylbertzenes

Compound	100% H_2_O		80% H_2_O		50% H_2_O		20% H_2_O		0% H_2_O	
	[in *C*_16_](M)	ln *K*_h/100%w_^a^	[in *C*_16_](M)	ln *K*_h/80%w_^a^	[in *C*_16_](M)	ln *K*_h/50%w_^a^	[in *C*_16_](M)	ln *K*_h/20%w_^a^	[in *C*_16_](M)	ln *K*_h/0%w_^a^
Biphenyl	3.91×10^−2^	9.65±0.02	3.91×10^−2^	8.43±0.07	3.91×10^−2^	5.57±0.04	3.91×10^−2^	3.06±0.01	3.91×10^−2^	0.53±0.01
Toluene	11.1×10^−2^	6.24±0.01	9.61×10^−2^	4.67±0.09	9.61×10^−2^	3.66±0.02	9.61×10^−2^	1.85±0.01	9.61×10^−2^	0.29±0.01
			0.02×10^−2^	5.01±0.10	0.02×10^−2^	3.29±0.08	0.02×10^−2^	1.49±0.03	0.67×10^−2^	0.31±0.01
									0.50×10^−2^	0.30±0.01
									0.33×10^−2^	0.29±0.01
Ethylbenzene	14.2×10^−2^	7.41±0.01	12.8×10^−2^	6.19±0.04	12.8×10^−2^	4.35±0.01	12.8×10^−2^	2.42±0.05	12.8×10^−2^	0.48±0.01
			0.02×10^−2^	5.98±0.07	0.02×10^−2^	4.01±0.07	0.02×10^−2^	1.93±0.03	0.87×10^−2^	0.45±0.01
									0.72×10^−2^	0.40±0.01
									0.46×10^−2^	0.39±0.01
		8.82±0.01	2.08×10^−2^	6.68±0.04	6.71×10^−2^	4.97±0.08	6.71×10^−2^	2.90±0.04	6.71×10^−2^	1.07±0.02
			0.01×10^−2^	7.15±0.06	0.01×10^−2^	4.86±0.06	0.01×10^−2^	2.58±0.02	0.87×10^−2^	0.93±0.01
									0.60×10^−2^	0.81±0.02
									0.46×10^−2^	0.80±0.01
*n*-Butylbenzene	7.55×10^−2^	10.50±0.03	9.57×10^−2^	8.04±0.02	9.57×10^−2^	5.62±0.03	9.57×10^−2^	3.52±0.05	9.57×10^−2^	1.50±0.03
			0.01×10^−2^	8.54±0.09	0.01×10^−2^	5.78±0.06	0.01×10^−2^	3.21±0.06	0.81×10^−2^	1.47±0.03
									0.47×10^−2^	1.20±0.02
									0.28×10^−2^	1.14±0.04
*n*-Pentylbenzene	7.18×10^−2^	11.61±0.20			7.18×10^−2^	6.26±0.02			7.18×10^−2^	1.86±0.08
*n*-Hexylbenzene	8.67×10^−2^	12.94±0.41			8.67×10^−2^	6.98±0.03			8.67×10^−2^	2.35±0.03

a The uncertainty is the standard deviation of the mean for three replicate measurements.

**Table 4:** Coefficients determined from regression analysis of the data of Hussam and Carr [19], according to the form^a^, $\ln\;\gamma_{a,x}=A+Bx_{b}+{Cx_{b}}^{2}+{Dx_{b}}^{3}$

Solute	*A*	*B*	C	*D*
Benzene	1.9767	2.9411	1.3497	1.4030
Toluene	2.2962	3.0698	2.9367	3.7922 ×10^−3^
Ethylbenzene	2.5489	3.7923	1.8357	1.7573
*n*-Propylbenzene	2.8088	4.2765	2.4203	1.2938
*n*-Butylbenzene	3.0462	5.0907	1.7578	2.3608

a ln γ_a,_*_x_* is the mole-fraction-based activity coefficient of the solute a, and *x*_b_ is the mole fraction of water in the water-methanol mixture.

**Table 5:** Comparison of activity coefficients determined from partition coefficients (25 °C) and from headspace analysis (30 °C)^a^

0% Water; *x*_b_=0					
Compound	ln*K*_h/0%w_	ln*V*_0%w_/*V*_h_^b^	lnγ_a(h),_*_x_*	lnγ_a(0%w),_*_x_*	
				Partition	Headspace^a^
Benzene	−0.07	−1.98	0.095	2.07	1.97
Toluene	0.29	−1.98	0.028	2.29	2.29
Ethylbenzene	0.39	−1.98	0.141	2.51	2.55
*n*-Propylbenzene	0.80	−1.98	0.130	2.91	2.80
*n*-Butylbenzene	1.14	−1.98	0.132	3.25	3.05

20 % Water; *x*_b_=0.3604					
Compound	ln*K*_h/20%w_	ln*V*_20%w_/*V*_h_^b^	lnγ_a(h),_*_x_*	lnγ_a(20%w),_*_x_*	
				Partition	Headspace^a^
Benzene	0.82	−2.20	0.095	3.12	3.28
Toluene	1.49	−2.20	0.028	3.72	3.78
Ethylbenzene	1.93	−2.20	0.141	4.27	4.24
*n*-Propylbenzene	2.58	−2.20	0.130	4.91	4.73
*n*-Butylbenzene	3.21	−2.20	0.132	5.54	5.22

50% Water; *x*_b_=0.6927					
Compound	ln*K*_h/50%w_	ln*V*_50%w_/*V*_h_^b^	lnγ_a(h),_*_x_*	lnγ_a(50%w),_*_x_*	
				Partition	Headspace^a^
Benzene	2.37	−2.46	0.095	4.93	5.13
Toluene	3.29	−2.46	0.028	5.78	5.83
Ethylbenzene	4.01	−2.46	0.141	6.62	6.64
*n*-Propylbenzene	4.86	−2.46	0.130	7.45	7.36
n-Butylbenzene	5.78	−2.46	0.132	8.38	8.20

80% Water; *x*_b_=0.9002					
Compound	ln*K*_h/80%w_	ln*V*_80%w_/*V*_h_^b^	lnγ_a(h),_*_x_*	lnγ_a(80%w),_*_x_*	
				Partition	Headspace^a^
Benzene	3.99	−2.67	0.095	6.76	6.74
Toluene	5.01	−2.67	0.028	7.71	7.44
Ethylbenzene	5.98	−2.67	0.141	8.79	8.73
*n*-Propylbenzene	7.15	−2.67	0.130	9.95	9.56
*n*-Butylbenzene	8.54	−2.67	0.132	11.34	10.78

100% Water; *x*_b_=1.0000						
Compound	ln*K*_h/100%w_	ln*V*_100%w_/*V*_h_^b^	lnγ_a(h),_*_x_*	lnγ_a(100%w),_*_x_*		
				Partition	Solub^c^	Headspace^a^
Benzene	4.77	−2.79	0.095	7.66	7.56	7.67
Toluene	6.24	−2.79	0.028	9.06	9.08	8.31
Ethylbenzene	7.41	−2.79	0.141	10.34	10.37	9.93
*n*-Propylbenzene	8.82	−2.79	0.130	11.74	11.77	10.80
*n*-Butylbenzene	10.50	−2.79	0.132	13.42	13.19	12.26

a Hussam and Carr [19].

b Ratio of molar volumes.

c Determined from aqueous solubility [2].
